# Poly[(μ_4_-5,7-di­hydro-1*H*,3*H*-dithieno[3,4-*b*:3′,4′-*e*]pyrazine-κ^4^ *N*:*N*′:*S*:*S*′)tetra-μ_3_-iodido-tetra­copper]: a three-dimensional copper(I) coordination polymer

**DOI:** 10.1107/S2414314620004010

**Published:** 2020-03-27

**Authors:** Tokouré Assoumatine, Helen Stoeckli-Evans

**Affiliations:** a Institute of Chemistry, University of Neuchâtel, Av. de Bellevax 51, CH-2000 Neuchâtel, Switzerland; b Institute of Physics, University of Neuchâtel, rue Emile-Argand 11, CH-2000 Neuchâtel, Switzerland; University of Toronto, Canada

**Keywords:** crystal structure, copper(I) iodide, pyrazine, pyrazine­thio­phane, three-dimensional coordination polymer, framework

## Abstract

The reaction of ligand 5,7-di­hydro-1*H*,3*H*-dithieno[3,4-*b*:3′,4′-*e*]pyrazine (**L**) with CuI lead to the formation of a three-dimensional coordination polymer, incorporating the well known [Cu_*x*_I_*x*_]_*n*_ staircase motif (*x* = 4). These polymer [Cu_4_I_4_]_*n*_ chains are linked *via* the N and S atoms of the ligand to form the three-dimensional coordination polymer poly[(μ_4_-5,7-di­hydro-1*H*,3*H*-dithieno[3,4-*b*:3′,4′-*e*]pyrazine-κ^4^ *N*:*N*′:*S*:*S*′)tetra-μ_3_-iodido-tetra­copper], [Cu_4_I_4_(C_8_H_8_N_2_S_2_)]_*n*_ (**I**). The asymmetric unit is composed of half a ligand mol­ecule, with the pyrazine ring located about a center of symmetry, and two independent copper(I) atoms and two independent I^−^ ions forming the staircase motif *via* centers of inversion symmetry. The framework is consolidated by C—H⋯I hydrogen bonds.

## Abstract 2

The reaction of ligand 5,7-di­hydro-1*H*,3*H*-dithieno[3,4-*b*:3′,4′-*e*]pyrazine with CuI lead to the formation of a three-dimensional coordination polymer.

## Graphical abstract

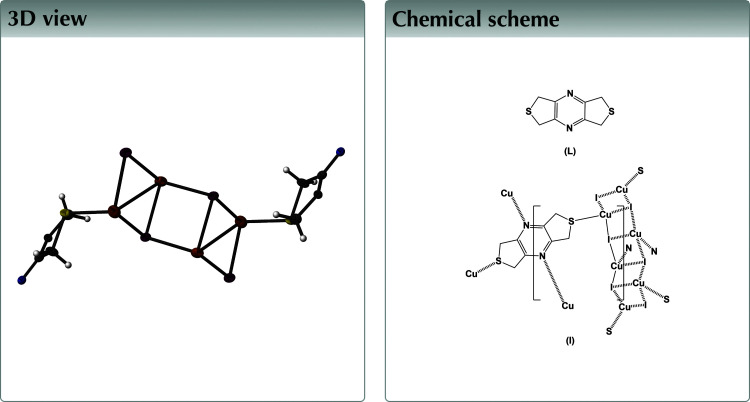

## Structure description

We have recently shown that the reaction of the ligand 5,7-di­hydro-1*H*,3*H*-dithieno[3,4-*b*:3′,4′-*e*]pyrazine (**L**), with silver(I) nitrate leads to the formation of a two-dimensional coordination polymer, with the silver atom coordinating only to the S atoms of the ligand so forming chains. The nitrato anion bridges two equivalent silver atoms and so generates the network structure (Assoumatine & Stoeckli-Evans, 2020*a* ▸). Ligand **L** is one of a series of pyrazine­thio­phanes synthesized to study their coordination chemistry with various transition metals (Assoumatine, 1999 ▸). The reaction of **L** with CuCl_2_ and CuBr_2_ lead to the formation of isostructural one-dimensional coordination polymers with the ligand coordinating to the copper atom *via* the N atoms only (Assoumatine & Stoeckli-Evans, 2020*b* ▸).

**Figure 1:**
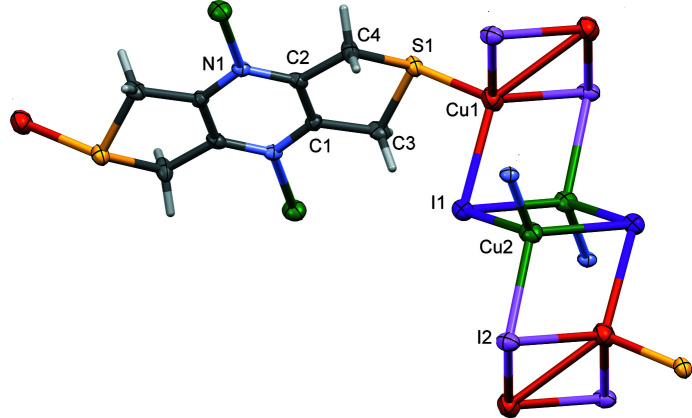
A partial view of the mol­ecular structure of complex **I**, with atom labelling for the atoms of the asymmetric unit. Displacement ellipsoids are drawn at the 30% probability level. Colour code: Cu1 orange, Cu2 green, I1 purple, I2 violet.

**Figure 2:**
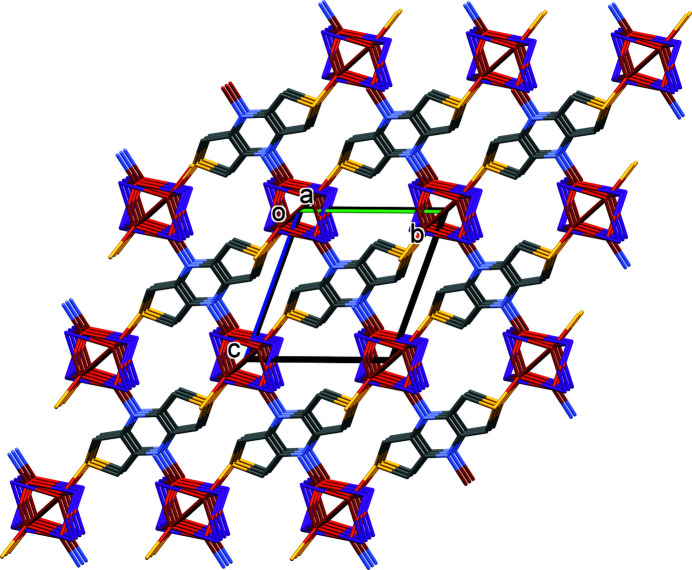
A view along the *a* axis of the crystal packing of complex **I**. For clarity, the H atoms have been omitted.

The reaction of **L** with CuI has lead to the formation of a three-dimensional coordination polymer, incorporating the well known [Cu_*x*_I_*x*_]_*n*_ staircase motif (*x* = 4; Fig. 1 ▸). The asymmetric unit is composed of half a ligand mol­ecule, with the pyrazine ring located about a center of symmetry, and two independent copper(I) atoms and two independent I^−^ ions forming the staircase motif *via* centers of inversion symmetry. The polymer [Cu_4_I_4_]_*n*_ chains are linked *via* the N and S atoms of the ligand to form the three-dimensional framework of complex **I** (Fig. 2 ▸).

In the ligand, the five-membered thio­phene rings are not planar, but have envelope configurations with the S atom as the flap. Atom S1 deviates by 0.5076 (14) Å from the mean plane of the four C atoms (C1–C4). This is considerably more than in the silver(I) nitrate two-dimensional coordination polymer or the ligand itself (Assoumatine & Stoeckli-Evans, 2020*a* ▸). In the former, the S atom deviates from the mean plane of the four C atoms by 0.170 (15) Å, and in the latter by only 0.0256 (8) Å.

Selected bond lengths and bond angles involving the copper(I) atoms in **I** are given in Table 1 ▸. In **I**, both copper(I) atoms are fourfold coordinate; the Cu⋯Cu distances are not considered as bonds. Hence, atom Cu1 has a fourfold CuSI_3_ coord­ination geometry with the fourfold index parameter τ_4_ = 0.91 (τ_4_ = 1 for a perfect tetra­hedral geometry, 0 for a perfect square-planar geometry and 0.85 for perfect trigonal–pyramidal geometry; Yang *et al.*, 2007 ▸). Atom Cu2 has a CuNI_3_ coordination geometry with a fourfold index parameter τ_4_ = 0.88. Hence, both atoms have similar distorted shapes, neither perfect tetra­hedral nor perfect trigonal–pyramidal. The Cu—N, Cu—S and Cu—I bond lengths are within normal values and are discussed below.

In the crystal of **I**, the three-dimensional structure is consolidated by C—H⋯I hydrogen bonds (Table 2 ▸).

There are less than 15 polymeric structures in the Cambridge Structural Database (CSD; Version 5.41, last update November 2019; Groom *et al.*, 2016 ▸) that concern pyrazine ligands and the [Cu_*x*_I_*x*_]_*n*_ staircase motif (see file S1 in the supporting information). The majority form two-dimensional coordination polymers with the pyrazine ligand bridg­ing the [Cu_*x*_I_*x*_]_*n*_ chains. For example, *catena*-[bis­(μ_3_-iodo)(μ_2_-pyrazine-*N,N′*)dicopper(I)] (CSD refcode AGIYEU01 at 203 K; Blake *et al.*, 1999 ▸) and *catena*-[bis­(μ_3_-iodo)(μ_3_-bis­(6-methyl­pyrazin-2-ylmeth­yl)thio­ether-*N,N′,N′′,S*)(μ_2_-iodo)­tri­copper(I)] (RABBUS at 123 K; Amoore *et al.*, 2003 ▸). In AGIYEU01, the copper atom has a CuNI_3_ fourfold coordin­ation geometry with a τ_4_ index parameter of 0.90. In the case of RABBUS, there are three copper(I) atoms. Two of the copper atoms have CuNI_3_ fourfold coordination geometries with τ_4_ index parameters of 0.92 and 0.89. The third copper atom has a CuNSI_2_ fourfold coordination geometry with a τ_4_ index parameter of 0.73. In comparison, the τ_4_ index parameters for the two copper atoms in **I** are 0.91 and 0.85.

In AGIYEU01, the Cu—N_pyrazine_ bond length is 2.028 (9) Å. In RABBUS the copper(I) atoms coordinate to the pyrazine N atoms and the S atom of the ligand. Here, the Cu—N_pyrazine_ bond lengths are 2.053 (4), 2.070 (4) and 2.071 (4) Å and the Cu—S bond length is 2.3574 (15) Å. In **I**, the Cu2—N^iv^ and Cu1—S1 bond lengths of 2.059 (4) and 2.3393 (16) Å, respectively, are very similar to those sited above. The Cu—I bond lengths involving the [Cu_*x*_I_*x*_]_*n*_ staircase motifs are very similar in all three compounds; they vary from 2.6131 (15) to 2.6485 (15) Å in AGIYEU01, from 2.5993 (7) to 2.8348 (7) Å in RABBUS, and from 2.6418 (8) to 2.7142 (9) Å in **I**. The Cu⋯Cu distances in **I**, Cu1—Cu1^i^ = 2.7355 (16) Å and Cu1—Cu2^ii^ = 2.9030 (14) Å, are similar to those in AGIYEU01 [2.7562 (19) Å] and RABUSS [2.6546 (8), 2.7565 (9) and 2.9256 (8) Å].

## Synthesis and crystallization

The synthesis and crystal structure of the ligand 5,7-di­hydro-1*H*,3*H*-dithieno[3,4-*b*:3′,4′-*e*]pyrazine (**L**), have been reported (Assoumatine & Stoeckli-Evans, 2020*a* ▸).

**Synthesis of compound I:** A solution of **L** (15 mg, 0.08 mmol) in CH_2_Cl_*2*_ (10 ml) was introduced into a 16 mm diameter glass tube and layered with MeCN (2 ml) as a buffer zone. Then a solution of CuI (15 mg, 0.08 mmol) in MeCN (5 ml) was added very gently to avoid possible mixing. The glass tube was sealed under an atmosphere of nitro­gen and left in the dark at room temperature for at least 2 weeks, whereupon deep-orange plate-like crystals of the title compound, (**I**), were isolated in the buffer zone. Analysis for C_8_H_8_N_2_S_2_Cu_4_I_4_ (*M*_r_ = 958.14 g mol^−1^): calculated (%): C 10.03, H 0.84, N 2.92, S 6.69; found (%): C 10.12, H 0.80, N 2.82, S 6.67. The IR spectrum is shown in Fig. S1 of the supporting information.

## Refinement

Crystal data, data collection and structure refinement details are summarized in Table 3 ▸. A Stoe IPDS-I one-circle diffractometer was used for the data collection at RT. With this instrument for the triclinic system often a small cusp of data is inaccessible; here 176 reflections which gives the alert *diffrn_reflns_laue_measured_fraction_full value (0.889) below minimum (0.95)*. The effect here appears to be limited if one compares the bond lengths in the structure of the pure ligand (Assoumatine & Stoeckli-Evans, 2020*a* ▸) with those of the ligand in complex **I**; they are the same within 3 s.u.s.

The residual electron-density peaks, Δρ_max_ = 1.73 e Å^−3^, and Δρ_min_ = −1.58 e Å^−3^, are located near the iodine atoms (within 0.80 to 1.13 Å). As stated by Spek (2018 ▸): ‘they can be inter­preted as absorption artefacts due to insufficient or incorrect correction for absorption’; the *T*_min_ and *T*_max_ values of 0.188 and 1.000, respectively, are rather extreme. However, in our experience high residual electron-density peaks are often observed for compounds containing heavy atoms such as iodine.

## Supplementary Material

Crystal structure: contains datablock(s) I, Global. DOI: 10.1107/S2414314620004010/lh4055sup1.cif

Structure factors: contains datablock(s) I. DOI: 10.1107/S2414314620004010/lh4055Isup2.hkl

CSD search. DOI: 10.1107/S2414314620004010/lh4055sup3.pdf

The IR spectrum. DOI: 10.1107/S2414314620004010/lh4055sup4.tif

CCDC reference: 1991908

Additional supporting information:  crystallographic information; 3D view; checkCIF report

## Figures and Tables

**Table 1:** Selected geometric parameters (Å, °)

Cu1—Cu1^i^	2.7355 (16)	I2—Cu1^ii^	2.6617 (10)
Cu1—Cu2^ii^	2.9030 (14)	Cu2—N1^iv^	2.059 (4)
Cu1—S1	2.3393 (16)	I1—Cu2^ii^	2.6786 (9)
I1—Cu1	2.6477 (9)	I1—Cu2	2.7142 (9)
I2—Cu1^iii^	2.6478 (11)	I2—Cu2	2.6418 (8)
			
S1—Cu1—I1	104.34 (4)	N1^iv^—Cu2—I2	107.06 (12)
S1—Cu1—I2^v^	108.74 (5)	N1^iv^—Cu2—I1^ii^	122.19 (13)
I1—Cu1—I2^v^	107.06 (3)	I2—Cu2—I1^ii^	113.30 (3)
S1—Cu1—I2^ii^	104.10 (5)	N1^iv^—Cu2—I1	110.69 (13)
I1—Cu1—I2^ii^	113.66 (3)	I2—Cu2—I1	108.98 (3)
I2^v^—Cu1—I2^ii^	117.98 (3)	I1^ii^—Cu2—I1	93.48 (3)

Symmetry codes: (i) 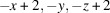 ; (ii) 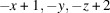 ; (iii) 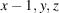 ; (iv) 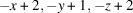 ; (v) 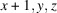 .

**Table 2:** Hydrogen-bond geometry (Å, °)

*D*—H⋯*A*	*D*—H	H⋯*A*	*D*⋯*A*	*D*—H⋯*A*
C3—H3*B*⋯I1	0.97	3.01	3.808 (6)	140
C3—H3*A*⋯I1^vi^	0.97	2.91	3.767 (5)	149
C4—H4*A*⋯I1^iv^	0.97	3.00	3.641 (5)	124

Symmetry codes: (iv) 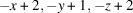 ; (vi) 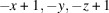 .

**Table 3:** Experimental details

Crystal data	
Chemical formula	[Cu_4_I_4_(C_8_H_8_N_2_S_2_)]
*M*_r_	958.04
Crystal system, space group	Triclinic, *P* 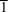
Temperature (K)	293
*a*, *b*, *c* (Å)	7.0082 (8), 8.378 (1), 8.8162 (10)
α, β, γ (°)	102.808 (13), 104.607 (13), 109.369 (13)
*V* (Å^3^)	445.35 (10)
*Z*	1
Radiation type	Mo *K*α
μ (mm^−1^)	11.87
Crystal size (mm)	0.50 × 0.35 × 0.13

Data collection	
Diffractometer	Stoe *IPDS1*
Absorption correction	Multi-scan (*MULABS*; Spek, 2020 ▸)
*T*_min_, *T*_max_	0.188, 1.000
No. of measured, independent and observed [*I* > 2σ(*I*)] reflections	3272, 1515, 1452
*R*_int_	0.038
(sin θ/λ)_max_ (Å^−1^)	0.612

Refinement	
*R*[*F* ^2^ > 2σ(*F* ^2^)], *wR*(*F* ^2^), *S*	0.040, 0.103, 1.10
No. of reflections	1515
No. of parameters	92
H-atom treatment	H-atom parameters constrained
Δρ_max_, Δρ_min_ (e Å^−3^)	1.73, −1.58

Computer programs: *EXPOSE*, *CELL* and *INTEGRATE* in *IPDS-I* (Stoe & Cie, 1998 ▸), *SHELXS97* (Sheldrick, 2008 ▸), *Mercury* (Macrae *et al.*, 2020 ▸), *SHELXL2018* (Sheldrick, 2015 ▸), *PLATON* (Spek, 2020 ▸) and *publCIF* (Westrip, 2010 ▸).

## full crystallographic data

### Crystal data

**Table:**

[Cu_4_I_4_(C_8_H_8_N_2_S_2_)]	*Z* = 1
*M**_r_* = 958.04	*F*(000) = 430
Triclinic, *P*1	*D*_x_ = 3.572 Mg m^−^^3^
*a* = 7.0082 (8) Å	Mo *K*α radiation, λ = 0.71073 Å
*b* = 8.378 (1) Å	Cell parameters from 5000 reflections
*c* = 8.8162 (10) Å	θ = 3.3–25.8°
α = 102.808 (13)°	µ = 11.87 mm^−^^1^
β = 104.607 (13)°	*T* = 293 K
γ = 109.369 (13)°	Plate, orange
*V* = 445.35 (10) Å^3^	0.50 × 0.35 × 0.13 mm

### Data collection

**Table:**

Stoe IPDS-1 diffractometer	1515 independent reflections
Radiation source: fine-focus sealed tube	1452 reflections with *I* > 2σ(*I*)
Plane graphite monochromator	*R*_int_ = 0.038
φ rotation scans	θ_max_ = 25.8°, θ_min_ = 3.3°
Absorption correction: multi-scan (MULABS; Spek, 2020)	*h* = −8→8
*T*_min_ = 0.188, *T*_max_ = 1.000	*k* = −10→10
3272 measured reflections	*l* = −10→10

### Refinement

**Table:**

Refinement on *F*^2^	Secondary atom site location: difference Fourier map
Least-squares matrix: full	Hydrogen site location: inferred from neighbouring sites
*R*[*F*^2^ > 2σ(*F*^2^)] = 0.040	H-atom parameters constrained
*wR*(*F*^2^) = 0.103	*w* = 1/[σ^2^(*F*_o_^2^) + (0.0717*P*)^2^ + 0.7287*P*] where *P* = (*F*_o_^2^ + 2*F*_c_^2^)/3
*S* = 1.10	(Δ/σ)_max_ < 0.001
1515 reflections	Δρ_max_ = 1.73 e Å^−^^3^
92 parameters	Δρ_min_ = −1.58 e Å^−^^3^
0 restraints	Extinction correction: (SHELXL2018; Sheldrick, 2015), Fc^*^=kFc[1+0.001xFc^2^λ^3^/sin(2θ)]^-1/4^
Primary atom site location: structure-invariant direct methods	Extinction coefficient: 0.016 (3)

### Special details

**Table:**

Geometry. All esds (except the esd in the dihedral angle between two l.s. planes) are estimated using the full covariance matrix. The cell esds are taken into account individually in the estimation of esds in distances, angles and torsion angles; correlations between esds in cell parameters are only used when they are defined by crystal symmetry. An approximate (isotropic) treatment of cell esds is used for estimating esds involving l.s. planes.

### Fractional atomic coordinates and isotropic or equivalent isotropic displacement parameters (Å^2^)

**Table:**

	*x*	*y*	*z*	*U*_iso_*/*U*_eq_	
I1	0.57925 (5)	0.17176 (5)	0.88315 (4)	0.0210 (2)	
I2	0.21064 (6)	0.27610 (5)	1.19497 (4)	0.0241 (2)	
Cu1	0.90704 (13)	0.07739 (12)	0.89969 (10)	0.0309 (3)	
Cu2	0.53098 (11)	0.18093 (10)	1.18047 (9)	0.0261 (2)	
S1	1.0313 (2)	0.16243 (17)	0.69503 (15)	0.0198 (3)	
N1	1.1948 (7)	0.6290 (6)	0.6269 (5)	0.0153 (9)	
C1	0.9013 (8)	0.3374 (7)	0.5075 (6)	0.0157 (10)	
C2	1.0949 (8)	0.4665 (7)	0.6316 (6)	0.0173 (10)	
C3	0.8095 (8)	0.1646 (8)	0.5351 (6)	0.0227 (12)	
H3A	0.759686	0.064557	0.433563	0.027*	
H3B	0.689123	0.157278	0.572613	0.027*	
C4	1.1768 (9)	0.4053 (7)	0.7736 (6)	0.0235 (11)	
H4A	1.146935	0.457309	0.869883	0.028*	
H4B	1.331493	0.439565	0.804763	0.028*	

### Atomic displacement parameters (Å^2^)

**Table:**

	*U*^11^	*U*^22^	*U*^33^	*U*^12^	*U*^13^	*U*^23^
I1	0.0227 (3)	0.0192 (3)	0.0175 (3)	0.00881 (19)	0.00387 (17)	0.00296 (18)
I2	0.0207 (3)	0.0222 (3)	0.0265 (3)	0.0103 (2)	0.00545 (18)	0.00384 (18)
Cu1	0.0322 (4)	0.0328 (5)	0.0296 (4)	0.0143 (4)	0.0077 (3)	0.0158 (3)
Cu2	0.0223 (4)	0.0226 (4)	0.0229 (4)	0.0062 (3)	0.0000 (3)	0.0025 (3)
S1	0.0224 (6)	0.0158 (7)	0.0185 (6)	0.0074 (5)	0.0034 (5)	0.0057 (5)
N1	0.0137 (18)	0.018 (2)	0.0127 (19)	0.0066 (17)	0.0032 (15)	0.0036 (16)
C1	0.015 (2)	0.015 (2)	0.016 (2)	0.005 (2)	0.0047 (18)	0.0048 (19)
C2	0.017 (2)	0.020 (3)	0.014 (2)	0.010 (2)	0.0030 (19)	0.004 (2)
C3	0.020 (3)	0.026 (3)	0.016 (2)	0.009 (2)	−0.0015 (19)	0.005 (2)
C4	0.027 (3)	0.016 (3)	0.018 (2)	0.004 (2)	−0.002 (2)	0.006 (2)

### Geometric parameters (Å, º)

**Table:**

Cu1—Cu1^i^	2.7355 (16)	S1—C3	1.825 (5)
Cu1—Cu2^ii^	2.9030 (14)	N1—C2	1.324 (7)
Cu1—S1	2.3393 (16)	N1—C1^v^	1.345 (6)
I1—Cu1	2.6477 (9)	C1—C2	1.400 (7)
I2—Cu1^iii^	2.6478 (11)	C1—C3	1.478 (8)
I2—Cu1^ii^	2.6617 (10)	C2—C4	1.509 (7)
Cu2—N1^iv^	2.059 (4)	C3—H3A	0.9700
I1—Cu2^ii^	2.6786 (9)	C3—H3B	0.9700
I1—Cu2	2.7142 (9)	C4—H4A	0.9700
I2—Cu2	2.6418 (8)	C4—H4B	0.9700
S1—C4	1.817 (6)		
			
Cu1—I1—Cu2^ii^	66.05 (3)	I2—Cu2—Cu1^ii^	57.14 (3)
Cu1—I1—Cu2	103.29 (3)	I1^ii^—Cu2—Cu1^ii^	56.46 (3)
Cu2^ii^—I1—Cu2	86.52 (3)	I1—Cu2—Cu1^ii^	105.41 (3)
Cu2—I2—Cu1^iii^	102.73 (3)	C4—S1—C3	93.7 (2)
Cu2—I2—Cu1^ii^	66.37 (3)	C4—S1—Cu1	107.46 (19)
Cu1^iii^—I2—Cu1^ii^	62.02 (3)	C3—S1—Cu1	109.11 (19)
S1—Cu1—I1	104.34 (4)	C2—N1—C1^v^	115.3 (4)
S1—Cu1—I2^vi^	108.74 (5)	C2—N1—Cu2^iv^	121.6 (3)
I1—Cu1—I2^vi^	107.06 (3)	C1^v^—N1—Cu2^iv^	123.0 (4)
S1—Cu1—I2^ii^	104.10 (5)	N1^v^—C1—C2	121.5 (5)
I1—Cu1—I2^ii^	113.66 (3)	N1^v^—C1—C3	122.6 (4)
I2^vi^—Cu1—I2^ii^	117.98 (3)	C2—C1—C3	115.9 (4)
S1—Cu1—Cu1^i^	123.23 (6)	N1—C2—C1	123.2 (5)
I1—Cu1—Cu1^i^	132.41 (5)	N1—C2—C4	122.9 (5)
I2^vi^—Cu1—Cu1^i^	59.24 (3)	C1—C2—C4	113.9 (5)
I2^ii^—Cu1—Cu1^i^	58.74 (4)	C1—C3—S1	105.0 (3)
S1—Cu1—Cu2^ii^	121.87 (5)	C1—C3—H3A	110.7
I1—Cu1—Cu2^ii^	57.49 (3)	S1—C3—H3A	110.7
I2^vi^—Cu1—Cu2^ii^	129.10 (4)	C1—C3—H3B	110.7
I2^ii^—Cu1—Cu2^ii^	56.48 (3)	S1—C3—H3B	110.7
Cu1^i^—Cu1—Cu2^ii^	94.20 (5)	H3A—C3—H3B	108.8
N1^iv^—Cu2—I2	107.06 (12)	C2—C4—S1	105.1 (4)
N1^iv^—Cu2—I1^ii^	122.19 (13)	C2—C4—H4A	110.7
I2—Cu2—I1^ii^	113.30 (3)	S1—C4—H4A	110.7
N1^iv^—Cu2—I1	110.69 (13)	C2—C4—H4B	110.7
I2—Cu2—I1	108.98 (3)	S1—C4—H4B	110.7
I1^ii^—Cu2—I1	93.48 (3)	H4A—C4—H4B	108.8
N1^iv^—Cu2—Cu1^ii^	143.81 (13)		
			
C1^v^—N1—C2—C1	−0.7 (8)	N1^v^—C1—C3—S1	164.0 (4)
Cu2^iv^—N1—C2—C1	176.2 (4)	C2—C1—C3—S1	−16.8 (6)
C1^v^—N1—C2—C4	−179.2 (5)	C4—S1—C3—C1	22.5 (4)
Cu2^iv^—N1—C2—C4	−2.3 (7)	Cu1—S1—C3—C1	132.4 (3)
N1^v^—C1—C2—N1	0.7 (8)	N1—C2—C4—S1	−164.6 (4)
C3—C1—C2—N1	−178.6 (5)	C1—C2—C4—S1	16.7 (6)
N1^v^—C1—C2—C4	179.3 (4)	C3—S1—C4—C2	−22.5 (4)
C3—C1—C2—C4	0.1 (7)	Cu1—S1—C4—C2	−133.8 (3)

Symmetry codes: (i) −*x*+2, −*y*, −*z*+2; (ii) −*x*+1, −*y*, −*z*+2; (iii) *x*−1, *y*, *z*; (iv) −*x*+2, −*y*+1, −*z*+2; (v) −*x*+2, −*y*+1, −*z*+1; (vi) *x*+1, *y*, *z*.

### Hydrogen-bond geometry (Å, º)

**Table:**

*D*—H···*A*	*D*—H	H···*A*	*D*···*A*	*D*—H···*A*
C3—H3*B*···I1	0.97	3.01	3.808 (6)	140
C3—H3*A*···I1^vii^	0.97	2.91	3.767 (5)	149
C4—H4*A*···I1^iv^	0.97	3.00	3.641 (5)	124

Symmetry codes: (iv) −*x*+2, −*y*+1, −*z*+2; (vii) −*x*+1, −*y*, −*z*+1.
