# Supplementary material for: Poly[(μ4-5,7-di­hydro-1H,3H-dithieno[3,4-b:3′,4′-e]pyrazine-κ4 N:N′:S:S′)tetra-μ3-iodido-tetra­copper]: a three-dimensional copper(I) coordination polymer
Source: IUCrdata. 2020 Mar 27;5(Pt 3):x200401. doi: 10.1107/S2414314620004010 (PMC9462195; doi:10.1107/S2414314620004010)

Search Overview

Search: search6

Date/Time done: Sat Mar 07 10:08:52 2020

Database(s): CSD version 5.41 (November 2019)

Restriction Info: No refcode restrictions applied

Filters: None

Percentage Completed: 100%

Number of Hits: 16

Summary of queries used. Search found structures that:

match

Query 2 **Query 3**

**Query 2**	Name **catena**
**Query 3**	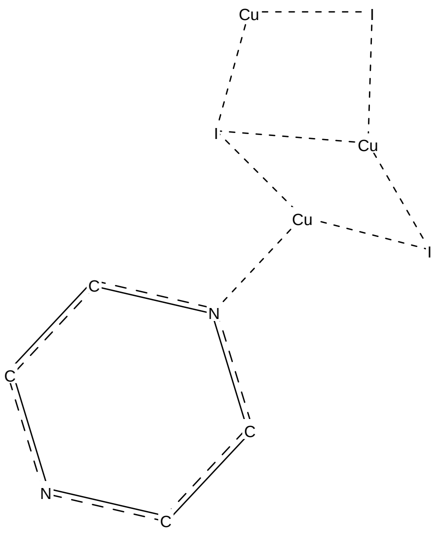

Search: search6 (Sat Mar 07 10:08:52 2020): Hits 1-4

AGIYEU01

Reference: A.J.Blake, N.R.Brooks, N.R.Champness, P.A.Cooke, M.Crew, A.M.Deveson, L.R.Hanton, P.Hubberstey, D.Fenske, M.Schroder (1999) *Crystal Engineering* ,**2,**181

Formula: (C_4 H_4 Cu_2 I_2 N_2)n

Compound Name: catena-(bis(μ_3-iodo)-(μ_2-pyrazine-N,N')-di-copper(i))

Space Group:	P-1	***Cell:***	***a***	4.134(0)	***b***	7.058(1)	***c***	8.041(1)
Space Group No.:	2	***(Å,°)***	α	109.72(3)	β	101.31(3)	γ	96.54(3)

R-Factor (%): 4.82 ***Temperature(K):*** 203 ***Density(g/cm³):*** 3.604

Parameters

Fragment 1
DIST1 (D) 2.028

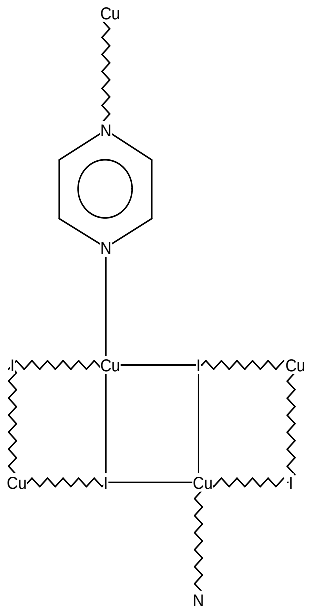

AGIYEU02

Reference: A.M.Goforth, M.D.Smith, H.-C.zur Loye (2003) *J.Chem.Cryst.* ,**33,**303

Formula: (C_4 H_4 Cu_2 I_2 N_2)n

Compound Name: catena-(bis(μ_3-Iodo)-(μ_2-pyrazine)-di-copper(i))

Space Group:	P-1	***Cell:***	***a***	4.176(0)	***b***	7.128(0)	***c***	8.128(0)
Space Group No.:	2	***(Å,°)***	α	109.61(0)	β	101.77(0)	γ	96.68(0)

R-Factor (%): 2.88 ***Temperature(K):*** 293 ***Density(g/cm³):*** 3.502

Parameters

Fragment 1
DIST1 (D) 2.053

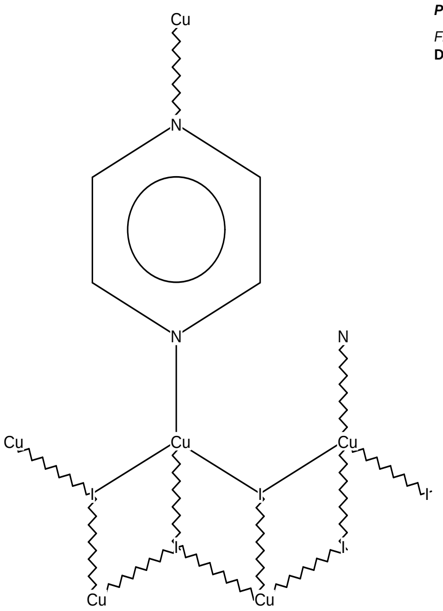

BASBAA

Reference: Jin-Cheng Wu, Liang Zhao, De-Xian Wang, Mei-Xiang Wang (2012) *Inorg.Chem.* ,**51,**3860

Formula: (C_{40} H_{40} Cu_5 I_5 N_{24})n

Compound Name: catena-(bis(μ_4-2,4,6,8-tetramethyl-2,4,6,8-tetraza-1,3,5,7(2,6)-tetrapyrazinacyclooctaphane)-bis(μ_3-iodo)-tris(μ_2-iodo)-penta-copper unknown solvate)

Synonym: catena-(bis(μ_4-tetramethylazacalix[4]pyrazine)-bis(μ_3-iodo)-tris(μ_2-iodo)-penta-copper unknown solvate)

Space Group:	Pmmn	***Cell:***	***a***	24.142(2)	***b***	35.780(7)	***c***	9.209(1)
Space Group No.:	59	***(Å,°)***	α	90.00	β	90.00	γ	90.00

R-Factor (%): 7.29 ***Temperature(K):*** 113 ***Density(g/cm³):*** 1.511

Parameters

Fragment 1
DIST1 (D) 2.000

Fragment 2
DIST1 (D) 2.049

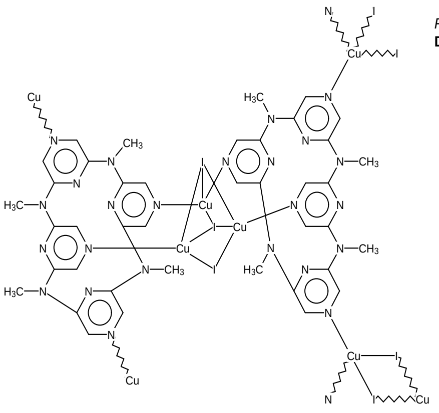

DEYNIG

Reference: Zhen-Zhen Wen, Xi-Ling Wen, Song-Liang Cai, Sheng-Run Zheng, Jun Fan, Wei-Guang Zhang (2013) *CrystEngComm* ,**15,**5359

Formula: (C_{10} H_8 Cu_3 I_2 N_4 O_5)n,H_2 O_1

Compound Name: catena-[bis(μ_3-Iodo)-bis(μ_2-pyrazine-2-carboxylato)-aqua-di-copper(i)-copper(ii) monohydrate]

Space Group:	Pbcn	***Cell:***	***a***	7.666(1)	***b***	31.951(7)	***c***	15.481(3)
Space Group No.:	60	***(Å,°)***	α	90.00	β	90.00	γ	90.00

R-Factor (%): 8.67 ***Temperature(K):*** 296 ***Density(g/cm³):*** 2.546

Parameters

Fragment 1
DIST1 (D) 2.090

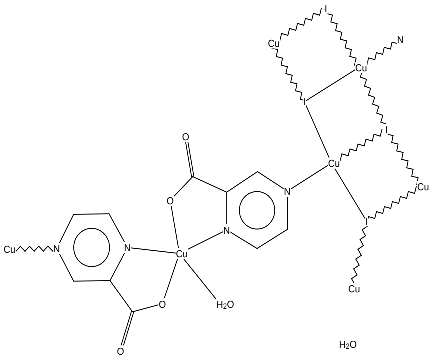

Search: search6 (Sat Mar 07 10:08:52 2020): Hits 5-8

DIVKUQ

Reference: Gao-Juan Cao, Zhi-Long Wang, Cheng Rong, Qing-Lu Li (2013) *Inorg.Chem.Commun.* ,**36,**163

Formula: $(C_{34}\ H_{24}\ Cu_2\ Dy_2\ I_2\ N_4\ O_{15})n$

Compound Name: catena-(bis(μ_4-Naphthalene-1,8-dicarboxylato)-bis(μ_3-iodo)-bis(μ_2-pyrazine-2-carboxylato)-triaqua-di-copper-di-dysprosium)

Space Group: Pbca ***Cell:*** ***a*** 8.160(0) ***b*** 29.750(1) ***c*** 30.838(2)
Space Group No.: 61 ***(Å,°)*** α 90.00 β 90.00 γ 90.00

R-Factor (%): 6.85 ***Temperature(K):*** 293 ***Density(g/cm³):*** 2.545

Parameters

Fragment 1
DIST1 (D) 2.058

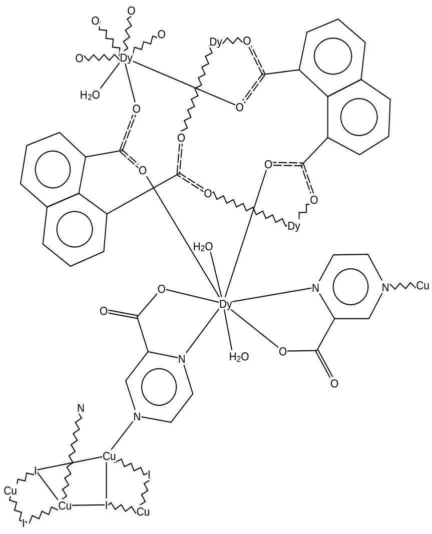

ECEVEO

Reference: J.H.Chong, M.J.MacLachlan (2006) *Inorg.Chem.* ,**45,**1442

Formula: $(C_{28}\ H_{17}\ Cu_4\ I_4\ N_7)n,n(C_2\ H_3\ N_1)$

Compound Name: catena-((μ_4-5,8,16,19,26,29-Hexa-azaoctacyclo(10.10.10.0^{2,11}.0^{4,9}.0^{13,22}.0^{15,20}.0^{23,32}.0^{25,30})dotriaconta-2(11),3,5,7,9,13(22),14,16,18,20,23(32),24,26,28,30-pentadecaene)-(μ_4-iodo)-(μ_3-iodo)-bis(μ_2-iodo)-(acetonitrile)-tetra-copper(i) acetonitrile clathrate)

Synonym: catena-((μ_4-Iodo)-(μ_4-tri-2,3-pyrazino(b,e,h)triptycene)-(μ_3-iodo)-bis(μ_2-iodo)-(acetonitrile)-tetra-copper(i) acetonitrile clathrate)

Space Group: P-1 ***Cell:*** ***a*** 9.837(0) ***b*** 11.970(0) ***c*** 15.862(0)
Space Group No.: 2 ***(Å,°)*** α 75.47(0) β 86.57(0) γ 67.83(0)

R-Factor (%): 2.60 ***Temperature(K):*** 173 ***Density(g/cm³):*** 2.490

Parameters

Fragment 1
DIST1 (D) 2.089

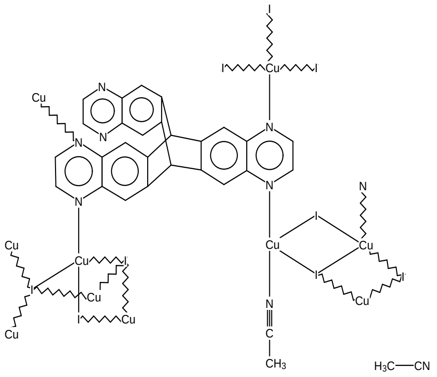

ECEVIS

Reference: J.H.Chong, M.J.MacLachlan (2006) *Inorg.Chem.* ,**45,**1442

Formula: $(C_{22}\ H_{14}\ Cu_2\ I_2\ N_2)n,n(C_2\ H_3\ N_1)$

Compound Name: catena-(bis(μ_3-Iodo)-(μ_2-5,8-di-azahexacyclo(10.6.6.0^{2,11}.0^{4,9}.0^{13,18}.0^{19,24})tetracosa-2(11),3,5,7,9,13,15,17,19,21,23-undecaene)-di-copper(i) acetonitrile clathrate)

Synonym: catena-((μ_3-Iodo)-(μ_2-2,3-pyrazino(b)triptycene)-di-copper(i) acetonitrile clathrate)

Space Group: P21/c ***Cell:*** ***a*** 9.157(1) ***b*** 31.760(4) ***c*** 8.246(0)
Space Group No.: 14 ***(Å,°)*** α 90.00 β 99.38(0) γ 90.00

R-Factor (%): 5.26 ***Temperature(K):*** 173 ***Density(g/cm³):*** 2.045

Parameters

Fragment 1
DIST1 (D) 2.057

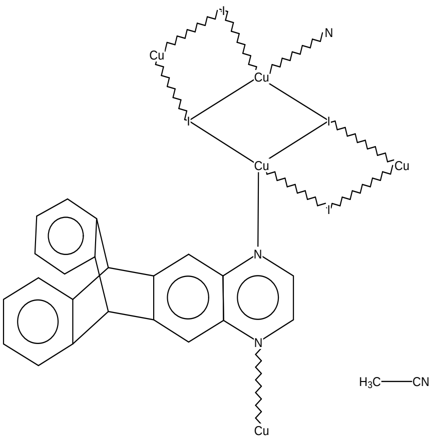

EMELOX

Reference: C.Nather, I.Jess, N.Lehnert, D.Hinz-Hubner (2003) *Solid State Sciences* ,**5,**1343

Formula: $(C_{12}\ H_{16}\ Cu_2\ I_2\ N_4)n$

Compound Name: catena-((μ_3-Iodo)-(μ_2-2-ethylpyrazine-N,N')-(μ_2-iodo)-(2-ethylpyrazine-N^4)-di-copper)

Space Group: P21/n ***Cell:*** ***a*** 10.434(0) ***b*** 14.803(0) ***c*** 11.109(0)
Space Group No.: 14 ***(Å,°)*** α 90.00 β 96.71(0) γ 90.00

R-Factor (%): 3.71 ***Temperature(K):*** 293 ***Density(g/cm³):*** 2.328

Parameters

Fragment 1
DIST1 (D) 2.080

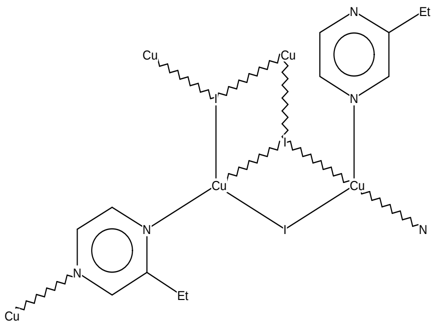

Search: search6 (Sat Mar 07 10:08:52 2020): Hits 9-12

LIDXUS

Reference: I.Jess, P.Taborsky, J.Pospisil, C.Nather (2007) *Dalton Trans.* ,2263

Formula: $(C_{12} H_{16} Cu_2 I_2 N_4)n$

Compound Name: catena-((μ_3-Iodo)-(μ_2-iodo)-(μ_2-2,3-dimethylpyrazine)-(2,3-dimethylpyrazine)-di-copper(i))

Space Group: P21/n ***Cell:*** ***a*** 9.851(0) ***b*** 12.848(0) ***c*** 13.584(0)
Space Group No.: 14 ***(Å,°)*** α 90.00 β 103.15(0) γ 90.00

R-Factor (%): 3.63 ***Temperature(K):*** 220 ***Density(g/cm³):*** 2.369

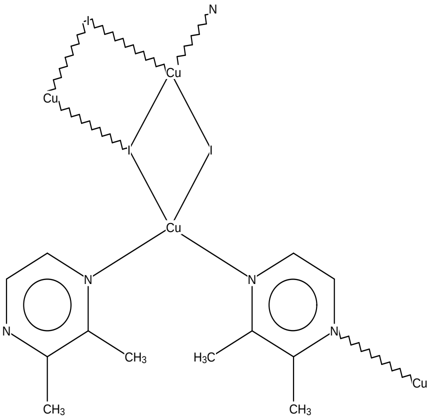

Parameters

Fragment 1
DIST1 (D) 2.062

Fragment 2
DIST1 (D) 2.050

MUHQOV

Reference: C.Nather, J.Greve, I.Jess (2002) *Solid State Sciences* ,**4,** 813

Formula: $(C_6 H_8 Cu_2 I_2 N_2)n$

Compound Name: catena-(bis(μ_3-Iodo)-(μ_2-2,5-dimethylpyrazine)-di-copper(i))

Space Group: P-1 ***Cell:*** ***a*** 4.294(0) ***b*** 8.181(1) ***c*** 8.215(1)
Space Group No.: 2 ***(Å,°)*** α 110.87(1) β 102.17(1) γ 94.54(1)

R-Factor (%): 2.31 ***Temperature(K):*** 293 ***Density(g/cm³):*** 3.126

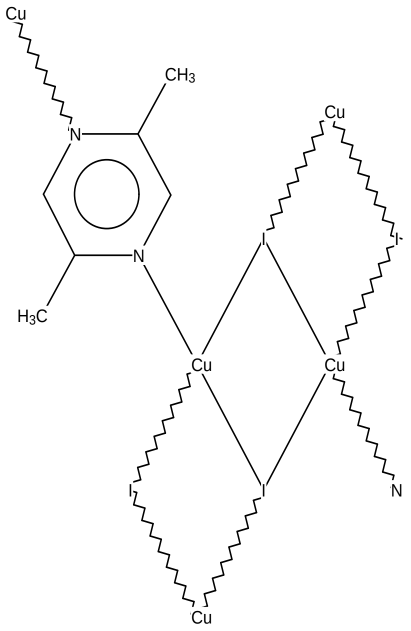

Parameters

Fragment 1
DIST1 (D) 2.058

MUHQOV01

Reference: N.Kitada, T.Ishida (2014) *CrystEngComm* ,**16,**8035

Formula: $(C_6 H_8 Cu_2 I_2 N_2)n$

Compound Name: catena-(bis(μ_3-Iodo)-(μ_2-2,5-dimethylpyrazine)-copper(i))

Space Group: P-1 ***Cell:*** ***a*** 4.297(1) ***b*** 8.220(3) ***c*** 8.247(3)
Space Group No.: 2 ***(Å,°)*** α 110.85(3) β 102.04(3) γ 94.82(3)

R-Factor (%): 5.90 ***Temperature(K):*** 293 ***Density(g/cm³):*** 3.097

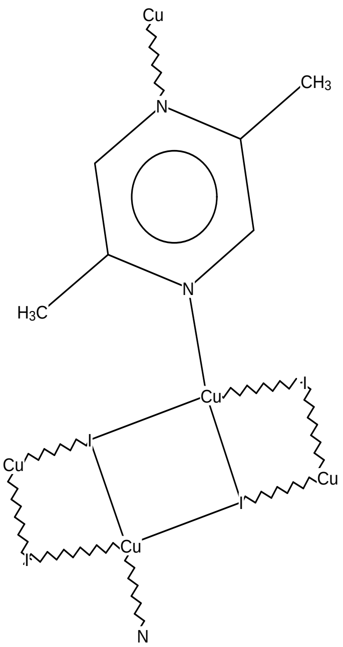

Parameters

Fragment 1
DIST1 (D) 2.066

RABBUS

Reference: J.J.M.Amoore, L.R.Hanton, M.D.Spicer (2003) *Dalton Trans.* ,1056

Formula: $(C_{12} H_{14} Cu_3 I_3 N_4 S_1)n$

Compound Name: catena-(bis(μ_3-Iodo)-(μ_3-bis(6-methylpyrazin-2-ylmethyl) thioether-N, N',N'',S)-(μ_2-iodo)-tri-copper(i))

Space Group: P-1 ***Cell:*** ***a*** 8.384(0) ***b*** 9.241(0) ***c*** 13.799(0)
Space Group No.: 2 ***(Å,°)*** α 87.94(0) β 86.21(0) γ 67.20(0)

R-Factor (%): 2.95 ***Temperature(K):*** 123 ***Density(g/cm³):*** 2.762

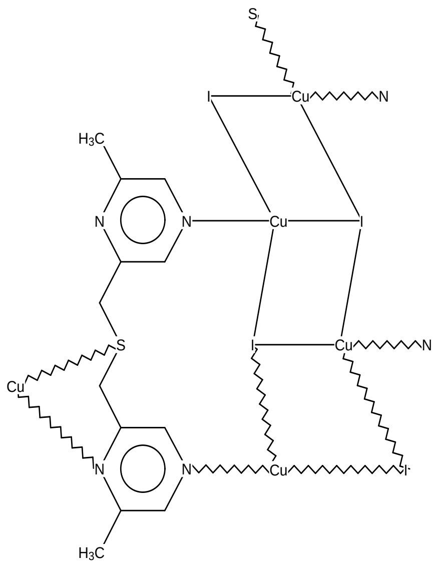

Parameters

Fragment 1
DIST1 (D) 2.070

Fragment 2
DIST1 (D) 2.053

Search: search6 (Sat Mar 07 10:08:52 2020): Hits 13-16

RUPTED

Reference: Yu-Jia Ding, Chun-Pei Zhang, Yong-Qing Wang, Xiao-Ming Lin, Ximiao Zhu, Da-Liang Zhang, Xian-Jian Duan, Yue-Peng Cai (2015) *CrystEngComm* ,**17,**6693

Formula: $(C_{10}\ H_8\ Cu_3\ I_2\ N_4\ O_5)n,0.5n(H_2\ O_1)$

Compound Name: catena-[bis(μ_3-Iodo)-bis(μ_2-pyrazine-2-carboxylato)-aqua-tri-copper hemihydrate]

Space Group:	Pbcn	***Cell:***	***a*** 7.668(0)	***b*** 31.909(3)	***c*** 15.427(1)
Space Group No.:	60	***(Å,°)***	α 90.00	β 90.00	γ 90.00

R-Factor (%): 4.22 ***Temperature(K):*** 296 ***Density(g/cm³):*** 2.526

Parameters

Fragment 1
DIST1 (D) 2.088

Fragment 2
DIST1 (D) 2.080

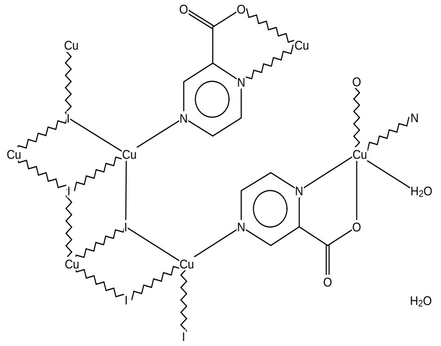

VEVGUY

Reference: P.M.Graham, R.D.Pike, M.Sabat, R.D.Bailey, W.T.Pennington (2000) *Inorg.Chem.* ,**39,**5121

Formula: $(C_8\ H_6\ Cu_2\ I_2\ N_2)n$

Compound Name: catena-(bis(μ_3-iodo)-(μ_2-quinoxaline-N,N')-di-copper(i))

Space Group:	P21/c	***Cell:***	***a*** 4.372(0)	***b*** 17.718(2)	***c*** 14.748(0)
Space Group No.:	14	***(Å,°)***	α 90.00	β 110.16(0)	γ 90.00

R-Factor (%): 6.18 ***Temperature(K):*** 295 ***Density(g/cm³):*** 3.165

Parameters

Fragment 1
DIST1 (D) 2.046

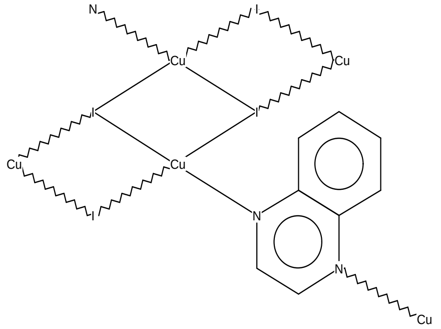

WURYUF

Reference: T.Hayashi, A.Kobayashi, H.Ohara, M.Yoshida, T.Matsumoto, Ho-Chol Chang, M.Kato (2015) *Inorg.Chem.* ,**54,**8905

Formula: $(C_{18}\ H_{18}\ Cu_4\ I_4\ N_6)n,n(C_7\ H_5\ N_1)$

Compound Name: catena-((μ_4-2,2',2''-Cyclohexane-1,3,5-triyltripyrazine)-tetrakis(μ_3-iodo)-tetra-copper benzonitrile solvate)

Space Group:	P21/c	***Cell:***	***a*** 14.765(5)	***b*** 17.932(6)	***c*** 16.285(5)
Space Group No.:	14	***(Å,°)***	α 90.00	β 105.68(0)	γ 90.00

R-Factor (%): 6.26 ***Temperature(K):*** 100 ***Density(g/cm³):*** 1.893

Parameters

Fragment 1
DIST1 (D) 2.037

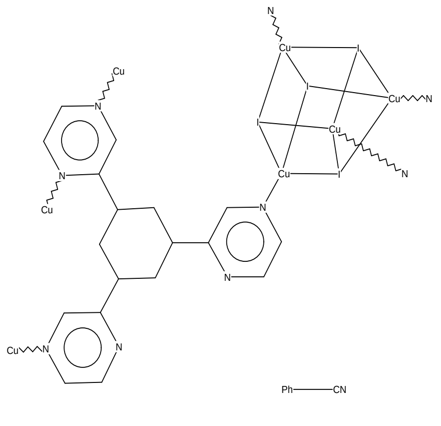

ZEBNUP

Reference: M.A.S.Goher, T.C.W.Mak (1995) *Polyhedron* ,**14,**2587

Formula: $(C_{10}\ H_8\ Cu_2\ I_2\ N_4\ O_4)n,3n(H_2\ O_1)$

Compound Name: catena(bis((μ_3-Iodo)-(pyridine-2-carboxylate)-copper(ii)) trihydrate)

Space Group:	P-1	***Cell:***	***a*** 6.813(1)	***b*** 8.273(1)	***c*** 16.719(2)
Space Group No.:	2	***(Å,°)***	α 93.44(1)	β 97.05(1)	γ 96.19(1)

R-Factor (%): 3.47 ***Temperature(K):*** 295 ***Density(g/cm³):*** 2.447

Parameters

Fragment 1
DIST1 (D) 2.063

Fragment 2
DIST1 (D) 2.053

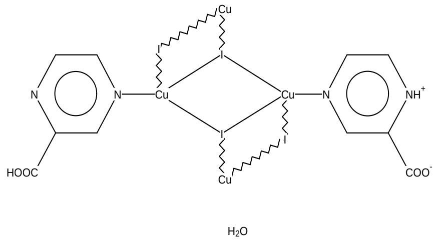

Supplement: Supplementary file 3 [file x-05-x200401-sup3.pdf]
