# Supplementary figures and images for: Poly[(μ4-5,7-di­hydro-1H,3H-dithieno[3,4-b:3′,4′-e]pyrazine-κ4 N:N′:S:S′)tetra-μ3-iodido-tetra­copper]: a three-dimensional copper(I) coordination polymer
Source: IUCrdata. 2020 Mar 27;5(Pt 3):x200401. doi: 10.1107/S2414314620004010 (PMC9462195; doi:10.1107/S2414314620004010)

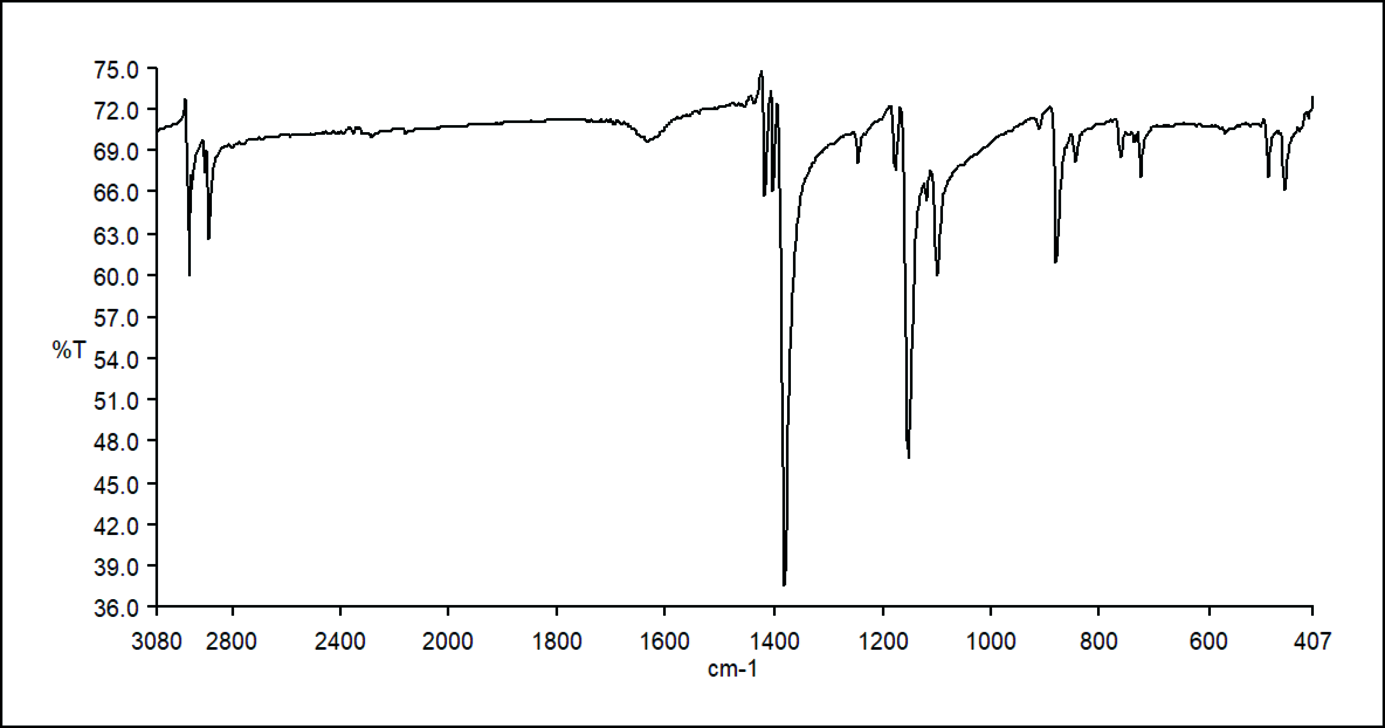

Supplement: Supplementary file 4 [file x-05-x200401-sup4.tif]
